# Operative management of acetabular fractures in the elderly: a case series

**DOI:** 10.1007/s00068-022-02129-0

**Published:** 2022-10-19

**Authors:** Michalis Panteli, Panayiotis Souroullas, Sushmith R. Gowda, James S. H. Vun, Anthony J. Howard, Nikolaos K. Kanakaris, Peter V. Giannoudis

**Affiliations:** 1 Academic Department of Trauma and Orthopaedics, School of Medicine, University of Leeds, Clarendon Wing, Level D, Great George Street, Leeds, LS1 3EX West Yorkshire UK; 2 Leeds Institute of Rheumatic and Musculoskeletal Medicine, University of Leeds, Leeds, UK; 3 Leeds General Infirmary, Leeds Orthopaedic and Trauma Sciences, University of Leeds, Leeds, UK; 4 Academic Department of Trauma and Orthopaedics, School of Medicine, Hull – York Medical School, Hull, UK; 5 Exeter Hip Unit, Royal Devon University Healthcare NHS Foundation Trust, Exeter, UK; 6 NIHR Leeds Biomedical Research Unit, Chapel Allerton Hospital, Leeds, UK

**Keywords:** Acetabular, Elderly, Operative, Total hip arthroplasty (THA), Complications

## Abstract

**Background:**

Our objective was to identify acetabular fractures in the elderly population (over 60 years of age), treated with open reduction and internal fixation (ORIF), and to examine their outcomes, primarily the risk for need for further surgery in the form of a total hip arthroplasty (THA), and factors associated with it. Additional outcomes such as infection, avascular necrosis (AVN) of the femoral head, and heterotopic ossification (HO) were also investigated.

**Methods:**

Following institutional review board (IRB) approval, a retrospective analysis of all consecutive patients presenting to a Level I Trauma Centre over a 13-years period (January 2003–February 2016) was conducted. Patients were excluded if their initial treatment was conservative or simultaneous ORIF with THA.

**Results:**

A total of 62 patients with an age of 71.5 ± 8.04 years were included (14 female; follow-up 54.2 months, range 1–195 months). Sixteen patients required a THA as a secondary procedure due to symptomatic post-traumatic arthritis (25.8%), five (8.1%) of whom having a THA within a year from the original trauma (three patients presenting with loss of reduction and two patients with early AVN). No associations with progression to THA were identified. Surgical approach (ilioinguinal) was the only factor associated with increased risk of development of HO (*p* = 0.010). The median post-operative survival following an acetabular fracture treated with ORIF was calculated at 90.1 months (95% CI 72.9–107.2).

**Conclusion:**

Acetabular fractures ORIF in the elderly, is a safe and reliable option. The relatively incidence of development of severe post-operative arthritis was 45.2%. Conversion to THA was 25.8%, with 8.1% having the arthroplasty procedure within a year of the original trauma surgery.

**Level of evidence:**

III.

## Introduction

In the United Kingdom alone, over 2000 acetabular fractures are reported annually, with the majority of them occurring in the elderly [1, 2]. These injuries typically involve a low energy mechanism, although high energy injuries secondary to road traffic collisions (RTCs) may also occur [3].

Pioneering work by Robert Judet and Emile Letournel [4, 5] has improved the understanding of complex pelvic anatomy and injury management, with excellent functional outcomes reported in up to 80% of operatively managed acetabular fractures [6]. Reconstitution of the articular surface of the acetabular weight-bearing portion has been demonstrated to be the most important predictor of a favourable outcome [5, 7–9]. ‘Burst’ type fractures involving superior segments and T-shaped posterior wall fractures have been associated with poorer outcomes, when compared to fractures involving the inferior acetabular area alone [5, 9–13]. This can be explained with the need for a difficult reduction manoeuvre, as well as the presence of a high rate of articular cartilage damage in these types of fractures [14].

In the elderly population, the different mechanisms of injury and the frequent presence of osteoporotic bone can lead to unique fracture patterns. These predominantly involve the anterior column, with posterior hemi-transverse and associated both column injuries. Achievement and continuous maintenance of fracture reduction can therefore be fraught with challenges and may correlate with joint destruction of these critical zones [15]. The goals of treatment in the elderly must be pain relief, early weight bearing and return to independent activities of daily living [16, 17], whilst treatment options include non-operative and operative management. Currently, there is no clear consensus on how to best manage acetabular fractures in the elderly.

The aim of this study was to report on patient demographics, fracture characteristics, prevalence of post-traumatic osteoarthritis and incidence of mortality in elderly patients (over 60 years of age) presenting with an acetabular fracture to a single Major Trauma Centre, managed with an open reduction and internal fixation (ORIF). Furthermore, we wished to evaluate potential associations with the need of conversion to a THA.

## Materials and methods

Following institutional review board (IRB) approval, a retrospective analysis of all consecutive patients presenting to a Level I Trauma Centre over a 13-years period (January 2003–February 2016) was conducted. Eligibility criteria included elderly patients over the age of 60 years presenting with an acetabular fracture requiring operative management (ORIF). Patients were excluded from the study if their initial management involved conservative treatment which either resulted in failure/complications necessitating surgical management, or if their care was transferred to other institutions. Patient receiving simultaneous ORIF and THA (at the same treatment episode) were also excluded.

All patients presented were initially managed by the Emergency Department (ED) team. High energy injuries were managed by the trauma multidisciplinary team (MDT) in accordance with the ATLS^®^ principles. Following the initial resuscitation, imaging of the pelvis was obtained, including plain radiographs and a computed tomography (CT) scan.

For the definitive management, patients were managed by the MDT (three fellowship trained pelvic surgeons, nursing staff, physiotherapists and occupational therapists), under a set protocol. All patients were positioned on a radiolucent table (OSI): supine in case of ilioinguinal or Stoppa approach; prone with traction in case of Kocher–Langenbeck approach. In cases of need for dual approach, this was performed in two stages (5–14 days between each stage, depending on physiological state of each patient). The type of fixation was left to the discretion of the operating surgeon. Post-operatively, patients followed a strict physiotherapy regime with 6 weeks of toe-touch weight bearing, followed by 6 weeks of partial weight bearing. Patients were followed-up at 2 and 6 weeks, 3, 6, 12 months, and yearly thereafter until resolution of symptoms or conversion to THA. The decision of conversion to THA was taken following a discussion between the patient and the treating physician.

### Data used for analysis

Information collected included patient demographics and comorbidities; mechanism of injury; time to theatre; operation details; complications (local and systemic); outcomes; length of hospital stay (LOS); and mortality. Fractures were classified according to the Letournel classification [4].

### Statistical analysis

Statistical analysis was performed using IBM SPSS Statistics version 28.0 software (SPSS Inc., Chicago, IL). The data set was analysed for normality prior to employing any statistical tests. All variables were independently assessed with a Pearson’s chi-squared test for statistical significance. A multivariable logistic regression model was used to adjust for confounders between independent factors and their potential association with conversion to THA and presence of HO. A *p* value < 0.05 was considered significant.

## Results

Out of 164 patients screened, a total of 62 (14 female) with a mean age 71.5 years (SD 8.04 years) fulfilled the eligibility criteria and were included in the final analysis (Table 1). The mean follow-up period was 54.2 months (range 1–195 months). The commonest mechanism of injury was a result of a road traffic accident (RTA), although the energy involved was described as low in 69.4% of the cases (*n* = 43). Majority of patients were direct admissions (*n* = 46; 72%), whereas the remaining patients were transferred from other institutions (*n* = 18; 28%), with an average of 6.7 days (SD 3.3 days) until transfer. Overall length of hospital stay (LOS) was 31.4 days (SD 22.5 days), with 39 patients (62.9%) requiring transfer to a higher dependency unit (HDU) or Intensive Care Unit (ICU), pre- or post-operatively or both (5.0 ± 4.9 days). Time of injury to definitive operation (ORIF), was 12.7 days (SD 31.0 days). The mean follow-up period was 54.2 months (SD 45.7 months).

**Table 1:** Patient demographics

	Number of patients
Gender	
Male	48
Female	14
Age at the time of operation (years) (mean ± SD)	71.5 ± 8.04
Cardiovascular disease	12
Cerebrovascular disease	9
Respiratory disease	7
Diabetes	8
Current/previous history of malignancy	7
Malignancy	7
Osteoporosis	4
Hypertension	9
Mechanism of Injury	
Fall from standing height	18
Fall from significant height	15
RTA	29
Unwitnessed fall	1
Seizure activity	1
Energy level of injury	
Low energy mechanism	43
High-energy mechanism	21
Hospital origin	
Transferred from another unit	18
Local MTC admission	46

Regarding fracture classification, 20 patients presented with an elementary type fracture pattern, compared to 44 patients displaying an associated fracture pattern (Tables 2 and 3). The commonest combination of associated type fractures was anterior/posterior column fractures (*n* = 17; 27.4%), followed by transverse type fractures (*n* = 7; 11.3%).

**Table 2:** Fracture patterns

	Anterior column involvement	Posterior column involvement	Anterior wall involvement	Posterior wall involvement	Transverse pattern	Posterior hemitransverse pattern	T-Shaped component	Sacro-Iliac Joint involvement	Associated Hip joint dislocation
Associated fracture pattern	28	27	1	17	11	5	1	1	7
Elementary fracture pattern	4	1	3	9	5	0	0	3	7

**Table 3:** Fracture pattern combinations by frequency of encounter

	Frequency (N)	Percent (%)
Posterior column	1	1.6
Both columns	16	25.8
Anterior wall	1	1.6
Anterior wall and anterior column	2	3.2
Posterior wall	1	1.6
Posterior wall and anterior column	1	1.6
Transverse	7	11.3
Anterior column and transverse	1	1.6
Posterior hemitransverse and anterior column	4	6.5
T-shaped	1	1.6
Posterior wall and transverse	5	8.1
Anterior column and SIJ involvement	1	1.6
Posterior column, anterior wall and posterior hemitransverse	1	1.6
Both columns and hip dislocation	1	1.6
Posterior wall with SIJ involvement	1	1.6
Both columns and articular impaction	2	3.2
Posterior wall involvement and hip dislocation	4	6.5
Posterior wall and transverse	2	3.2
Posterior wall, transverse and Articular impaction	2	3.2
Both column involvement and hip dislocation	2	3.2
Posterior wall involvement, hip dislocation and articular impaction	4	6.5
Posterior wall involvement, transverse, hip dislocation and articular impaction	2	3.2
Total	62	100.0

Fracture fixation combinations used to stabilise these injuries are presented in Table 4. The commonest plate combination used was one consisting of an anterior column plate, a quadrilateral plate fixation plate and cannulated screws to augment the fixation, usually in the anterior to posterior column direction.

**Table 4:** Fracture fixation combination pattern

Frequency		Percent (%)
Anterior column plate, cannulated AP screw and quadrilateral plate	10	16.1
Posterior column plate and cannulated AP screw	9	14.5
Posterior column plate	7	11.3
Anterior column plate and cannulated screw	5	8.1
Cannulated screw fixation	4	6.5
Anterior column plate	3	4.8
Anterior column plate and quadrilateral plate	3	4.8
Anterior column plate, cannulated screw and SIJ fixation	3	4.8
Posterior column plate and posterior wall plate	2	3.2
Anterior column plate and medial wall plate	2	3.2
MUA and traction	1	1.6
Posterior column plate	1	1.6
Quadrilateral plate	1	1.6
Posterior wall plate and cannulated screw	1	1.6
SIJ fixation and cannulated screw	1	1.6
Anterior and posterior column plates	1	1.6
Anterior and posterior column plates, posterior wall plate	1	1.6
Anterior column plate, quadrilateral plate and iliac wing plate	1	1.6
Anterior column plate, quadrilateral plate and SIJ fixation	1	1.6
Anterior and posterior column plates, quadrilateral plate	1	1.6
Anterior and posterior column plates, quadrilateral plate, iliac wing plate	1	1.6
Anterior column plate, cannulated screw and iliac wing plate	1	1.6
Anterior column plate, quadrilateral plate, Cannulated screws and iliac wing plate	1	1.6
Anterior and posterior column plates, quadrilateral plate and cannulated screw	1	1.6
Total	62	100.0

A single surgical approach was used in most of the cases, with ilioinguinal approach being the commonest approach utilised (*n* = 33, 53.2%) (Table 5). The Kocher–Langenbeck approach was used in 33% of the cases (*n* = 21), whereas a dual approach was deemed necessary in five patients (6.5%). An isolated percutaneous approach (cannulated screws) was utilised in five patients (8.1%). The mean operative length of time was 203.8 min (SD 101 min).

**Table 5:** Surgical approaches

	Frequency	Percent (%)
Ilioinguinal approach	33	53.2
Kocher–Langenbeck approach	20	32.3
Percutaneous approach	5	8.1
Combined approach	4	6.5
Total	62	100.0

**Fig. 1:**
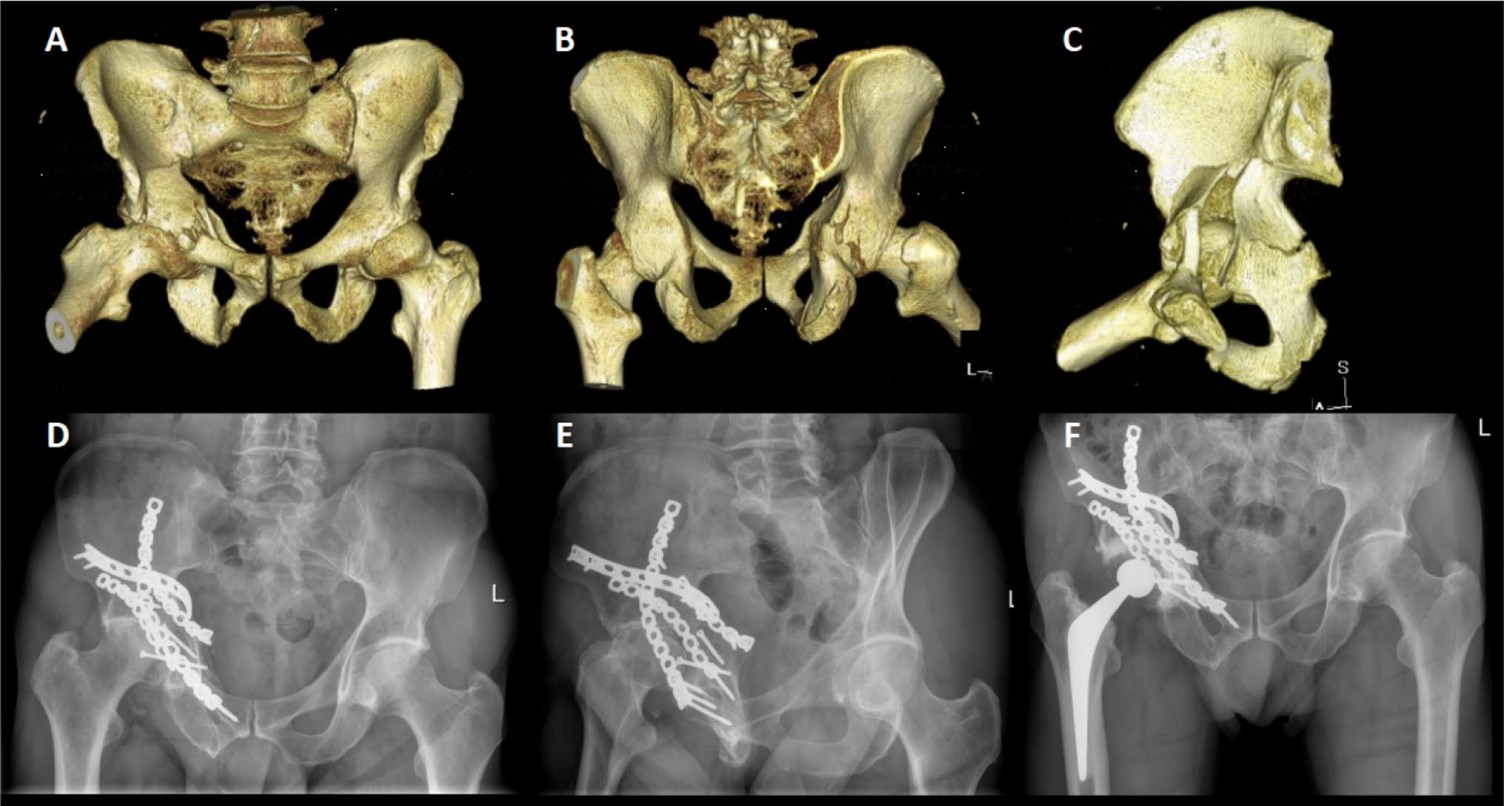
Case report: 69-year-old patient presenting with transverse posterior wall fracture following an RTA. **A** 3D reconstruction view (anterior view). **B** 3D reconstruction view (posterior view). **C** 3D reconstruction view (lateral view). **D** Plain radiograph (antero-posterior) demonstrating AVN of the femoral head, with associated loss of joint space and subchondral sclerosis. **E** Plain radiograph (Judet Iliac oblique view) demonstrating AVN of the femoral head, with loss of joint space and subchondral sclerosis, at one-year follow-up post ORIF. **F** Plain radiograph (antero-posterior) at five-year follow-up post THA

Concomitant injuries in this patient group were frequent, with limb fractures being the commonest (*n* = 30; 48.4%), followed by thoracic wall injuries (*n* = 11; 17.7%) (Table 6). Regarding post-operative complications, HO was the most observed complication (*n* = 9; 14.5%) (only one patient had surgical removal of the HO lesion followed by radiotherapy; the remaining patients were managed conservatively), whereas infection was reported in three patients (these were managed with intravenous antibiotics, with surgical washout and debridement deemed necessary in one patient) (Table 7). Nerve associated lesions were recorded in eight patients (12.9%): sciatic nerve injury secondary to the original trauma in three patients (4.8%), two of which had full recovery whilst one patient had persistent motor deficit; another five patients had neurapraxia of the lateral cutaneous nerve of the thigh, which resolved in all patients within the first year post-operatively (all patients had ilioinguinal approach fixation of their fractures). Early AVN of the femoral head was evident in two patients (3.2%) (Fig. 1).

**Table 6:** Concomitant injuries

	Count	Percentage (%)
Thoracic wall injury	11	17.7
Other limb fractures	30	48.4
Spinal injury	5	8.1
Abdominal injury	5	8.1
Head injury	4	6.5

**Table 7:** Post-operative complications

	Count	Percentage (%)
Heterotopic ossification	9	14.5
Avascular necrosis	2	3.2
Surgical site infection	3	4.8
Nerve injury		
Sciatic nerve	3	4.8
Lat. Cutaneous nerve	5	8.1
Loss of reduction (within 12 weeks from surgery)	3	4.8
Osteoarthritic changes at final follow-up		
Good preservation of joint space	20	32.3
Mild/moderate OA changes	14	22.6
Severe OA changes	28	45.2

**Fig. 2:**
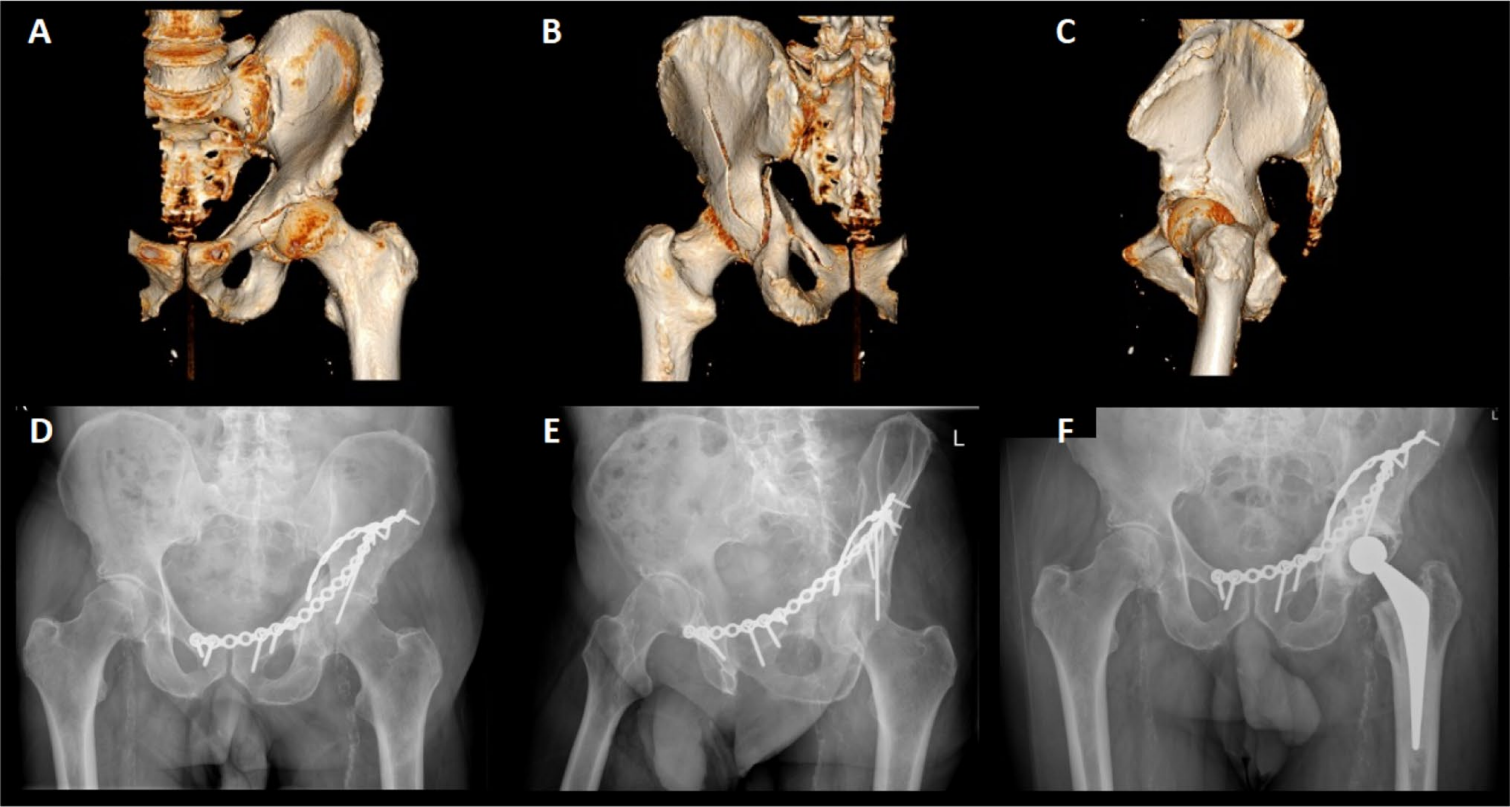
Case report: 69-year-old patient presenting with transverse posterior wall fracture following an RTA. **A** 3D reconstruction view (anterior view). **B** 3D reconstruction view (posterior view). **C** 3D reconstruction view (lateral view). **D** Plain radiograph (antero-posterior) demonstrating OA of the hip joint, with associated loss of joint space and subchondral sclerosis. **E** Plain radiograph (Judet obturator oblique view) demonstrating AVN of the femoral head, with loss of joint space and subchondral sclerosis, at one-year follow-up post ORIF. **F** Plain radiograph (antero-posterior) at five-year follow-up post THA

In three patients (4.8%), reduction was lost, with protrusion becoming evident in the early post-operative radiographs (within 12 weeks of fixation). In one of these patients, significant lack of compliance to protected weight bearing was thought to be the cause, whilst reduced bone density was considered the cause in the remaining two patients. Overall, a total of 16 patients (25.8%) required a THA (Fig. 2). Of these, five patients (8.1%) had the arthroplasty procedure within a year of the original trauma surgery (three patients presenting with loss of reduction and two patients with early AVN).

Independent factors including age at the time of injury and surgery, gender, fracture pattern energy level and presence of a hip dislocation at the time of the injury were examined to assess their association with the need to undergo THA as a secondary delayed procedure. None of the aforementioned factors were found to be statistically significantly associated with an increasing probability of requiring a THA at a secondary stage. These were further examined into a multivariable logistic regression analysis model to adjust for confounders between the independent factors (Table 8). This analysis did not reveal any statistically significant associations either. Additional factors were independently examined to investigate their association with development of HO as a significant post-operative complication (Table 9). The use of the ilioinguinal was the only factor associated with a statistically significant increased risk of developing HO (*p* = 0.010; OR 32.06; 95% CI 2.27–468.79).

**Table 8:** Independent analysis of potential factors associated with an increasing probability of requiring a THA at a secondary stage

	*p* value	OR	95% CI	
			Lower	Upper
Age at time of the injury	0.847	1.010	0.910	1.122
Energy level of injury (low)	0.492	1.700	0.374	7.728
Gender (male)	0.345	0.444	0.082	2.396
Surgical approach (ilioinguinal)	0.353	2.068	0.447	9.569
Fracture pattern (associated)	0.440	1.792	0.408	7.869
Presence of traction preoperatively	0.098	3.559	0.790	16.030
Associated hip dislocation	0.218	0.278	0.036	2.131

**Table 9:** Independent analysis of potential factors associated with an increasing probability of developing heterotopic ossification

	*p* value	OR	95% CI	
			Lower	Upper
Age at the time of the injury	0.218	1.098	0.946	1.274
Energy level of injury (low)	0.998	< 0.001	< 0.001	0.003
Gender (male)	0.765	1.475	0.115	18.868
Surgical approach (ilioinguinal)	**0.010**	32.606	2.268	468.789
Fracture pattern (associated)	0.379	0.324	0.026	3.980
Traction used preoperatively	0.998	1.003	0.078	12.959
Associated hip dislocation	0.758	0.652	0.043	9.920

Significant parameters are presented in bold (*p* < 0.05)

Further Kaplan–Meier survival analysis was performed to look at the effect of fracture pattern (Associated Vs Elementary) and mechanism of injury energy (High Vs Low energy) on the cumulative probability of requiring a THR.

**Fig. 3:**
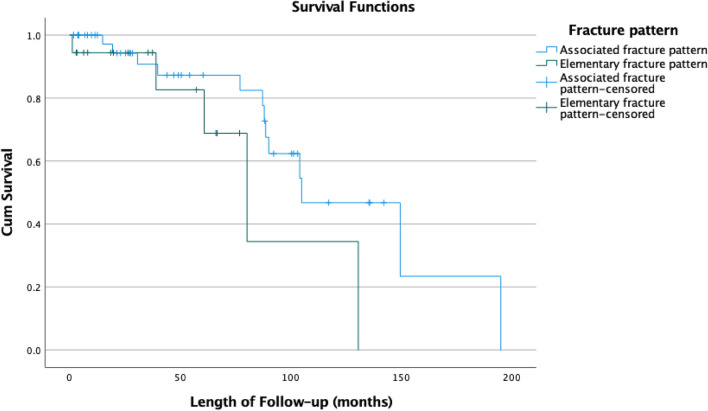
Kaplan–Meier curve investigating progression to THA, depending on fracture pattern

**Fig. 4:**
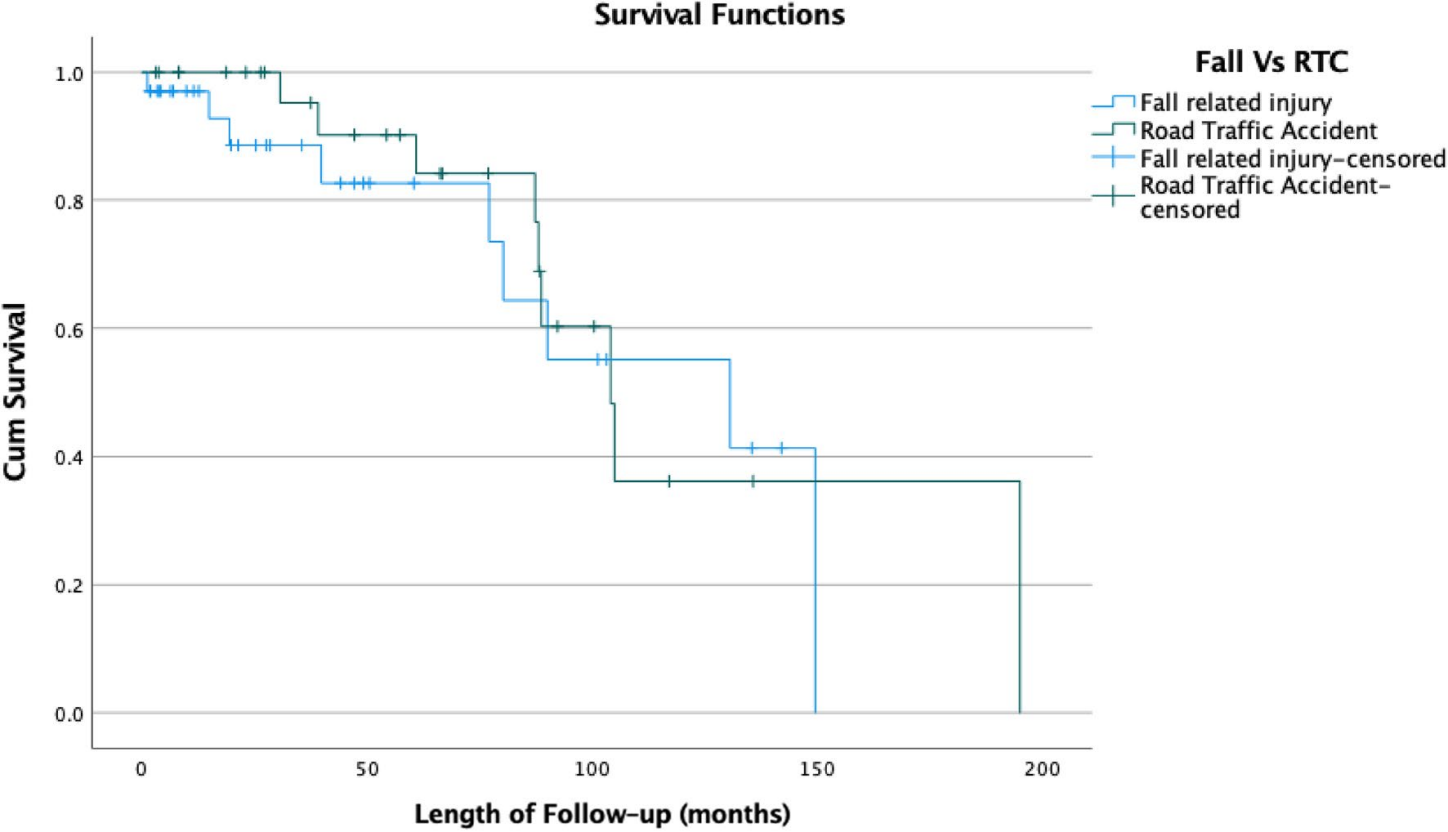
Kaplan–Meier curve investigating progression to THA, depending on mechanism of injury

The median time to a THR in patients with an elementary fracture pattern was 80.3 months (95% CI 51.9–108.6) compared to 105 months (95% CI 68.1–141.9) in those with an associated fracture pattern. There was however no statistically significant difference in the cumulative survival probability in requiring a THA in future [Log-rank test (Mantel-Cox): *p* = 0.104] (Fig. 3). Mechanism of injury (high versus low energy) also demonstrated no association [Log-Rank test (Mantel-Cox) *p* = 0.607] (Fig. 4).

**Fig. 5:**
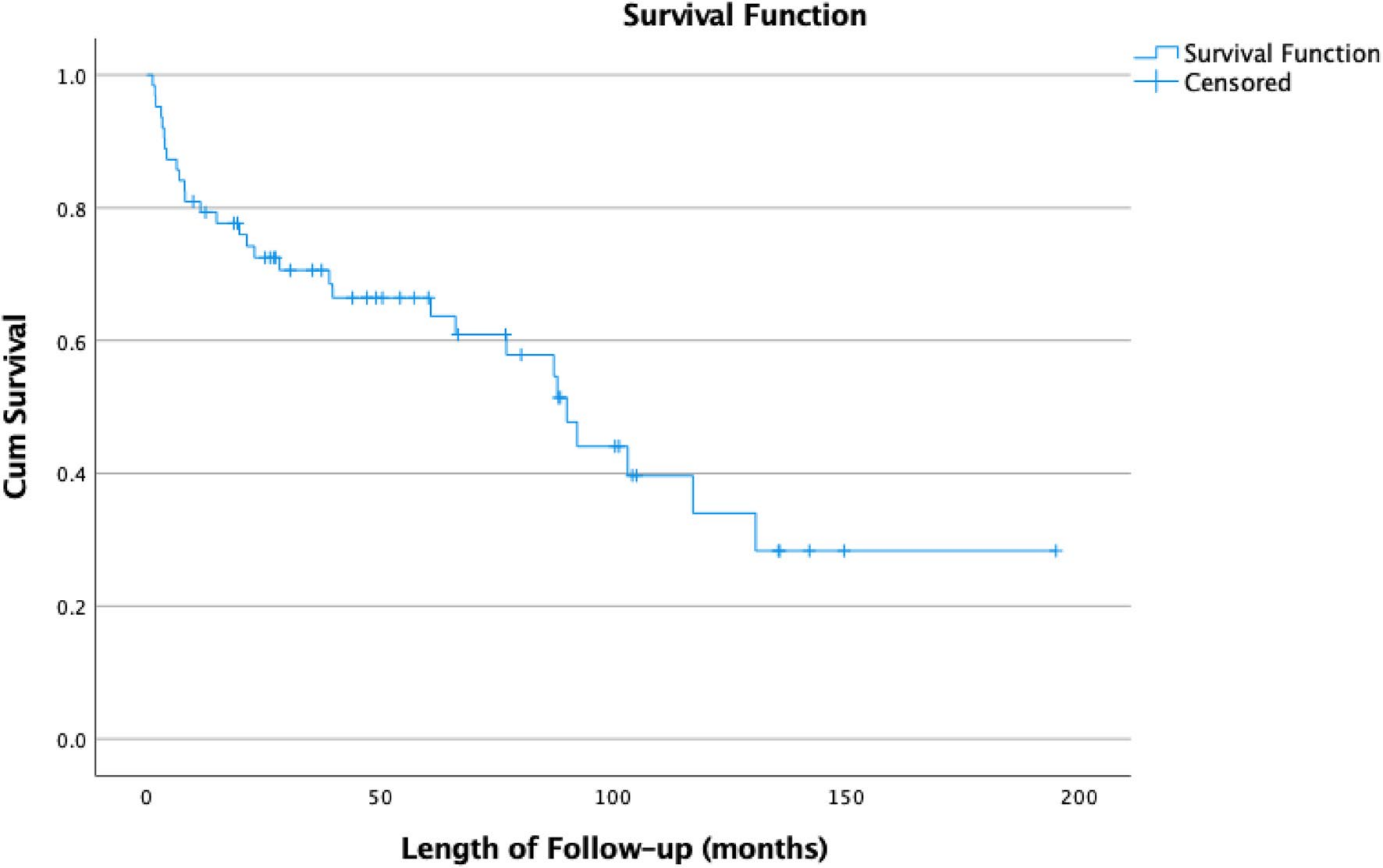
Kaplan–Meier curve investigating mortality following an acetabular fracture in our population

Finally, the median post-operative survival following an acetabular fracture treated with ORIF was calculated at 90.1 months (95% CI 72.9–107.2) (Fig. 5).

## Discussion

In the herein study, we evaluated the medium- to long- term clinical outcomes of ORIF for the treatment of acetabular fractures in the elderly. The mean age of our cohort was 71.5 years, whilst the patients included were frail with multiple co-morbidities. The rate of conversion to THA following acetabular ORIF was 25.8% (*n* = 16), which is comparable to the re-operation rates from studies by Khoshbin et al. and Navarre et al. [18, 19]. In terms of additional post-operative complication rates, nine patients developed HO, two patients developed early AVN, surgical site infection (SSI) was reported in three patients, loss of reduction in three patients and five patients had sustained a post-operative nerve palsy (all involved the lateral cutaneous nerve of the thigh, following an ilioinguinal approach). A study by Weaver et al. identified a trend for higher re-operation rates in the ORIF group in terms of a secondary THA, however, it did point out a higher complication rate in terms of infection/dislocation when compared to THAs performed for osteoarthritic changes [20]. Although SSI was noted in our patient cohort, only one patient required a re-operation as a result.

In our series, all 16 patients undergoing a THR received cemented components, with only one patient having a constrained cup because of compliance issues and high risk of dislocation. The complication rate of our THA group is comparable with that of the literature, with one patient undergoing a revision because of deep infection, one patient having chronic dislocation secondary to aseptic cup loosening (managed conservatively as not fit for revision surgery) and one patient sustaining a minimally displaced Vancouver B2 periprosthetic fracture that was managed conservatively.

Using a multivariate analysis model, we did not identify any factors associated with progression to THA following acetabular ORIF. This contradicts the findings from a study by Rollman et al. which suggested that increasing age by one year, fracture displacement by 1 mm, involvement of the posterior wall, or contusion/impaction of the femoral head were all independently associated with increased risk of need for a subsequent THA procedure [21]. The above study, however, did not have any age restrictions in their inclusion criteria and therefore is not necessarily applicable to the elderly population. The only association we noted was the increased risk of development of HO following ilioinguinal approach.

Elderly patients have multiple comorbidities and as such operative times should be kept to a bare minimum, as should surgical procedures. In this series, we demonstrate that neither fracture pattern and complexity, nor mechanism of injury had any effect on the risk for need for a THA as a delayed procedure. We also carried out a literature review to evaluate the latest outcomes of treatment which revealed six studies published between 2018 and 2022 (Tables 10, 11) [18, 19, 22–25]. Our series provides the most detailed record of the post-operative period in operatively treated acetabular fractures in the elderly over a long follow up period. Open reduction and internal fixations of acetabular fractures is, therefore, a safe and viable option in the treatment of such fractures in the elderly, in the right kind of patient, with outcomes which are as comparable in terms of performance as a patient treated with a simultaneous THA [18, 19, 22–25].

**Table 10:** Literature review of patients presenting with acetabular farctures, treated with open reduction and internal fixation (ORIF)

Paper	Year of publication	Number of patients in the study (> 60 years)	Age (mean/SD/range) (years)	Follow-up period (months)	Gender (female/%)	Post-operative Complications										
						Mortality	Dislocation	Re-operation (infection)	Re-operation (secondary THA)	Loosening	Re-operation (Wound Dehiscence)	Nerve palsy	VTE requiring treatment	Fracture	ANV	Heterotopic ossification
Borg et al	2018	14	72.2 (50–89)	36	10 (37%)	7.4% (24 months)	1	0	9	0	0	0	0	N/A	1	2
Khoshbin et al	2020	273	69.9 (SD 7.8)	82.7	75 (27.5%)	7.7% (90 days)	N/A	8	53	N/A	N/A	N/A	6	0	N/A	N/A
Navarre et al	2020	72	69.7 (SD 7.9)	18	19 (23.7%)	8.8% (18 months)	N/A	N/A	17	N/A	N/A	N/A	N/A	N/A	N/A	N/A
Nicol et al	2020	85	76 (SD 8)	12	12 (13.7%)	26% (mortality among 23 patients included in the final analysis)	0	1	14	1	0	0	0	1	N/A	N/A
Manson et al	2022	22	70.7 (SD 8.7)	12	8 (36%)	10.6% (12 months)	0	2	9	0	0	1	0	0	N/A	1
Panteli et al	2022	62	71.5 (SD 8.4)	54.2	14 (22.5%)	9.7% (12 months) 14.5% (24 months)	0	0	16	0	0	5	0	0	2	9

**Table 11:** Literature review of patients presenting with acetabular farctures, treated with open reduction and internal fixation (ORIF) and total hip arthroplasty (THA) on the same sitting

Paper	Year of publication	Number of patients in the study (> 60 years)	Age (mean/SD/range) (years)	Follow-up period (months)	Gender (female/%)	Post-operative complications										
						Mortality	Dislocation	Re-operation (infection)	Re-operation (secondary THA)	Loosening	Re-operation (Wound Dehiscence)	Nerve palsy	VTE requiring treatment	Fracture	ANV	Heterotopic ossification
Chen et al	2020	8	77 (59–89)	12	3 (37.5%)	N/A	N/A	N/A	N/A	N/A	1	N/A	N/A	N/A	N/A	5
Navarre et al	2020	8	69.7 (SD 7.9)	18	19 (23.7%)	8.8% (18 months)	N/A	N/A	17	N/A	N/A	N/A	N/A	N/A	N/A	N/A
Nicol et al	2020	12	81 (SD 7)	12	12 (13.7%)	26% (mortality among 23 patients included in the final analysis	1	1	0	0	0	0	0	0	N/A	N/A
Manson et al	2022	25	72.8 (SD 8)	12	7 (28%)	10.6% (12 months)	0	0	1	0	1	0	0	0	N/A	0

Like many other reports there are some notable limitations in the herein study such as the lack of reporting of PROMs (patient reported outcome measures), as well as the overall relatively small sample size which may well be the cause for masking some of the true effects of these factors providing a more definitive answer. It does nonetheless signify the need for a well-designed multicentre RCT (randomised controlled trial) comparing three arms (conservative versus ORIF versus simultaneous ORIF + THA) with clinical and patient reported outcome measures to answer the question.

### Conclusion

Open reduction and internal fixation of acetabular fractures in the elderly is a safe and reliable option, not related to an increased risk of complications. The relatively incidence of development of severe post-operative arthritis was 45.2%. Conversion to THA was 25.8%, with 8.1% having the arthroplasty procedure within a year of the original trauma surgery secondary to loss of reduction and early AVN. Even though HO was the most commonly observed complication (*n* = 9; 14.5%), only one patient required re-operation to address this.
